# Histone deacetylase 6 inhibition prevents hypercholesterolemia-induced erectile dysfunction independent of changes in markers of autophagy

**DOI:** 10.1093/sexmed/qfae096

**Published:** 2025-01-09

**Authors:** Colin M Ihrig, McLane M Montgomery, Yohei Nomura, Mitsunori Nakano, Deepesh Pandey, Justin D La Favor

**Affiliations:** Department of Health, Nutrition, and Food Sciences, Florida State University, Tallahassee, FL 32306, United States; Department of Health, Nutrition, and Food Sciences, Florida State University, Tallahassee, FL 32306, United States; Department of Cardiovascular Surgery, Saitama Medical Center, Jichi Medical University, Saitama 330-8507, Japan; Department of Cardiovascular Surgery, Tokyo Metropolitan Bokutoh Hospital, Tokyo, 130-8575, Japan; Vascular Biology Center and Department of Medicine, Medical College of Georgia at Augusta University, Augusta, GA 30912, United States; Department of Health, Nutrition, and Food Sciences, Florida State University, Tallahassee, FL 32306, United States

**Keywords:** sexual health, HDAC6, tubacin, PCSK9, endothelial, H_2_S, mitophagy, cholesterol, atherosclerosis, cystathionine gamma-lyase

## Abstract

**Background:**

Erectile dysfunction is a condition with a rapidly increasing prevalence globally with a strong correlation to the increase in obesity and cardiovascular disease rates.

**Aim:**

The aim of the current study is to investigate the potential role of tubacin, a histone deacetylase 6 (HDAC6) inhibitor, in restoring erectile function in a hypercholesterolemia-induced endothelial dysfunction model.

**Methods:**

Thirty-nine male C57Bl/6 J mice were divided into 3 groups. Two groups were administered an adeno-associated virus encoding for the gain of function of proprotein convertase subtilisin/kexin type 9 (PCSK9) and placed on a high-fat diet (HFD) with 1.25% cholesterol added for 18 weeks in order to induce a prolonged state of hypercholesterolemia. One of the PCSK9 groups received daily intraperitoneal injections of the HDAC6 inhibitor tubacin, while the other 2 groups received daily vehicle injections. Erectile function was assessed through measurement of intracavernosal pressure and mean arterial pressure during cavernous nerve stimulation, as well as assessment of agonist-stimulated ex vivo relaxation of the corpus cavernosum (CC). Western blotting was performed from CC tissue samples.

**Outcomes:**

Erectile and endothelial functions were assessed, as well as protein markers of mitochondrial dynamics, mitophagy, and autophagy.

**Results:**

Erectile function was impaired in the HFD + PCSK9 group throughout the entire voltage range of stimulation. However, the HFD + PCSK9 mice that were treated with tubacin experienced significant restoration of erectile function at the medium and high voltages of nerve stimulation. Similarly, ex vivo CC relaxation responses to acetylcholine and the cystathionine γ-lyase (CSE) substrate L-cysteine were reduced in the vehicle-treated HFD + PCSK9 mice, both of which were restored in the HFD + PCSK9 mice treated with tubacin. Corpus-cavernosum protein expression of CSE was significantly elevated in the tubacin-treated HFD + PCSK9 mice relative to both other groups. There were no significant differences observed in any of the protein markers of mitochondrial dynamics, mitophagy, or autophagy investigated.

**Clinical translation:**

Histone deacetylase 6 inhibition may protect against erectile and endothelial dysfunction associated with hypercholesterolemia.

**Strengths and limitations:**

This was the first study to investigate HDAC6-specific inhibition for treatment of erectile dysfunction. A study limitation was the exclusive focus on the CC, rather than structure and function of the pre-penile arteries that may develop a substantial atherosclerotic plaque burden under hypercholesterolemic conditions.

**Conclusions:**

Tubacin may prevent hypercholesterolemia-induced erectile dysfunction through a hydrogen sulfide–related mechanism unrelated to regulation of mitophagy or autophagy.

## Introduction

Erectile dysfunction (ED) is the inability to achieve or maintain an erection capable of sexual performance. Erectile dysfunction has also been presented as a possible indicator of a more systemic vascular dysfunction, specifically endothelial dysfunction.^1^^,^^2^ Erectile and endothelial dysfunction are both associated with cardiovascular disease (CVD),^3^ while atherosclerosis represents a major cause of CVD progression.^4^ Atherosclerosis is characterized by chronic inflammation leading to increased vascular damage and endothelial dysfunction. This chronic inflammation results from a multitude of pathologies including mitochondrial dysfunction and reactive oxygen species (ROS) production, as well as increased proliferation, migration, and senescence of vascular smooth muscle.^5^ Increased circulating oxidized LDL-cholesterol is associated with an increase in overall risk factors for CVD. These metabolic changes that occur lead to the inflammatory cascade.

Hydrogen sulfide (H_2_S) has become of increasing interest as a gasotransmitter joining the endogenous gaseous signaling molecules nitric oxide (NO) and carbon monoxide (CO).^6^^,^^7^ Hydrogen sulfide can act as a regulator of vascular tone working as a hyperpolarization factor with crosstalk between production of H_2_S and NO. Hydrogen sulfide also plays a regulatory role in the proliferation and migration of vascular smooth muscle cells aiding in maintaining vascular homeostasis and blunting vascular disease progression.^8^ Hydrogen sulfide is produced enzymatically via cysteine metabolism by 3 enzymes, cystathionine-γ-lyase (CSE), cystathionine-β-synthase (CBS), and 3-mercaptopyruvate sulfotransferase (3MST). Cystathionine-γ-lyase is the predominant H_2_S-producing enzyme in the vasculature and plays a role in maintenance of vascular functioning.^9^ Cystathionine-γ-lyase and H_2_S have also been shown to be beneficial in reduction of endothelial inflammation and atherogenic development and progression.^10^ Histone deacetylase 6 (HDAC6) is classified as a class IIb histone deacetylase (HDAC) and plays a regulatory role in cellular homeostasis.^11^ Histone deacetylases, along with histone acetyltransferase (HAT), work in balance to regulate transcription via posttranslational modifications.^12^ Increased activity of HDAC6 has shown to increase lipid-induced endothelial dysfunction in vitro as well as decrease CSE expression in aortic endothelial cells.^13^ Inhibitors of HDAC6 have shown to both increase CSE protein expression as well as blunt the down regulation of CSE by angiotensin II.^14^

Maintenance of the smooth muscle of the corpus cavernosum (CC) and the underlying vasculature is a critical component for maintenance of healthy erectile functioning. Autophagy, a form of lysosomal degradation, serves this function not only acting to eliminate cellular waste and damaged organelles but also as a stimulator of cellular homeostasis.^15^ Histone deacetylase 6 has shown to play a significant role in the regulation of autophagy via its post-translational modifications. Histone deacetylase 6 downregulates the autophagy pathway via its inhibition of transcription factor EB (TFEB). Inhibition of HDAC6 has been shown to be beneficial at restoration of normal autophagic cellular cleaning.^16^ Autophagy is also responsible for influencing the phenotypic transition of smooth muscle in response to stressors.^17^ Phenotypic changes in vascular smooth muscle are induced by stress and vascular injury including in lipid-rich environments. Disrupted autophagy may cause an imbalance in this phenotypic switching, leading to progression of atherosclerosis and vascular pathology.^18^

Mitophagy is a selective form of autophagy performed by the mitochondria in response to damage from stressors such as increased ROS generation. Under physiological conditions, mitophagy can efficiently clear these damaged mitochondria and maintain cellular homeostasis. However, when the stressors placed on the mitochondria become too great, the process of mitophagy becomes disordered and results in more damaged mitochondria remaining in the cell. These damaged mitochondria without being effectively eliminated go from anti-inflammatory to pro-inflammatory, adding to the growing stress.^19^ Another major stressor for mitochondrial functioning and overall inflammatory levels is oxidized LDL. Systemically elevated oxidized LDL is detrimental to mitophagy and increases the prevalence of endothelial cell damage and apoptosis.^20^ This increase in mitochondrial dysfunction contributes to the inflammatory burden that leads to pathologic vascular development and atherogenesis. In preventing mitochondrial dysfunction and increasing stability of mitochondrial biogenesis, both CSE and H_2_S have been shown to play a multifactorial role. Maintenance of H_2_S production is critical for maintenance of homeostatic mitochondrial functioning leading to increased mitochondrial stability and increased ability to buffer physiologic stress.^21^ Histone deacetylase 6 may also play a vital role in maintenance of mitophagy via its regulatory role of BNIP3 protein expression.^22^ In this study, we sought to address the following questions: (1) Does HDAC6 inhibition preserve erectile function and penile endothelial function in a model of hypercholesterolemia?, (2) Does HDAC6 inhibition upregulate CSE expression in the hypercholesterolemic corpus cavernosum?, and (3) Does HDAC6 inhibition alter markers of autophagy and mitophagy in the hypercholesterolemic corpus cavernosum?

## Methods

### Experimental animals and study design

Male C57Bl/6 J mice were purchased from Jackson Laboratories (Bar Harbor, ME, USA). Mice were maintained in a pathogen-free animal housing facility with ad libitum access to food and drinking water. At 8 weeks of age, mice were administered a single tail-vein injection of 1 × 10^11^ vector genomes of adeno-associated virus (AAV) encoding a gain-of-function mutant (D377Y) form of proprotein convertase subtilisin/kexin type 9 (PCSK9).^23^ These mice then received a high-fat diet (HFD, 40% energy from fat) with 1.25% cholesterol added (#D12108C, Research Diets, New Brunswick, NJ, USA) for 18 weeks. This hypercholesterolemic mouse model has been described previously. Hepatic LDL receptor expression becomes depleted, and mice develop extreme increases in non-HDL cholesterol levels within 1 week following PCSK9 AAV injection.^24^^,^^25^ Two groups (n = 13 per group) of these mice received a daily intraperitoneal injection of either vehicle (dimethylsulfoxide [DMSO]) or the HDAC6 inhibitor tubacin (0.5 mg/kg; Enzo Life Sciences, New York, NY, USA). A separate set of control mice (n = 13) received a single tail-vein injection of saline at 8 weeks of age and was maintained on a standard chow diet for 18 weeks thereafter. All experimental procedures were approved by the Institutional Animal Care and Use Committee of Johns Hopkins University.

### Erectile function assessment

Erectile function was assessed through measurement of intracavernosal pressure (ICP) and mean arterial pressure (MAP) as described previously.^26^ Briefly, mice were anesthetized with an intraperitoneal injection of 90 mg/kg ketamine and 10 mg/kg xylazine. The left carotid artery and left crus were cannulated with polyethylene tubing filled with 100 U/mL of heparinized saline connected to pressure transducers (ADInstruments, Sydney, Australia), allowing for the continuous measurement of MAP and ICP through connection to a PowerLab data acquisition system (ADInstruments) via a bridge amplifier (#FE221, ADInstruments) with LabChart Pro software (ADInstruments). The cavernous nerve was stimulated with bipolar electrodes via a square pulse stimulator (Grass Instruments, Quincy, MA, USA) at a frequency of 10 Hz with a 5 ms pulse width for 60 s stimulation periods, separately at 1, 2, and 4 V of stimulation. Mice were sacrificed 10 minutes following the final stimulation by double thoracotomy and exsanguination of the vena cava. Erectile function was assessed by the peak ICP-to-MAP ratio and the area under the curve (AUC) of the ICP tracing during stimulation normalized to MAP (AUC/MAP), which represent the ability to achieve and maintain an erection, respectively.

### Ex vivo vascular reactivity of the corpus cavernosum

In a separate set of mice from the erectile function assessment, mice were deeply anesthetized with an intraperitoneal injection of 90 mg/kg ketamine and 10 mg/kg xylazine and sacrificed by double thoracotomy and exsanguination of the vena cava. Penile tissue was removed under a dissection microscope, and the penile shaft was separated from the glans penis. The penile shafts were placed in ice-cold Krebs solution of the following composition (in mM): NaCl 130, KCl 4.7, KH_2_PO_4_ 1.18, MgSO_4_ 1.18, NaHCO_3_ 14.9, dextrose 5.6, CaCl_2_ 1.56, and EDTA 0.03 dissolved in distilled water. All components of the Krebs solution were purchased from Sigma Aldrich (St. Louis, MO, USA). The urethra, dorsal vein, and connective tissues were carefully excised from the penile shaft in chilled Krebs solution. The CC tissue was mounted in a DMT 820MS muscle strip myograph system (Danish MyoTechnology, Aarhus, Denmark) for isometric tension measurement and recording with LabChart software (ADInstruments).^27^ Tissues were bathed in Kreb’s solution maintained at 37 °C and continuously aerated with a 95% O_2_ and 5% CO_2_ mixture. Tissues were allowed to equilibrate for 1 hour, stretched to a resting tension of 4 mN, followed by an additional 1 hour of equilibration. Tissue viability and contractile function were tested with high-potassium (120 mM) Krebs solution, with KCl substituted for NaCl. Following successive washes with Krebs solution to achieve a stable resting tension, tissues were pre-constricted with 10 μM phenylephrine (PE), followed by cumulative dose–responses of acetylcholine (ACh: 0.001-3.0 μM) to test endothelial function, sodium nitroprusside (SNP: 0.001-3.0 μM) to test endothelium-independent relaxation, and the CSE substrate L-cysteine (L-Cys: 0.1 μM-1.0 mM). Tissues were washed thrice successively with Krebs solution over 30 minutes following the dose–response protocol for each agonist. All relaxations were calculated as a percentage restoration to the resting tension from the PE pre-constricted value.

### Immunoblotting analysis

Immediately following sacrifice of mice that underwent erectile function assessment, penile tissue was harvested from the base to the proximal glans, from which the corpus spongiosum, dorsal vein, and connective tissue were quickly stripped off. The CC was quickly rinsed in ice-cold PBS, blood was removed from the tissue, and the tissue snap frozen in liquid nitrogen and stored at -80 °C until processing. Tissues were homogenized and immunoblotting analysis was performed as described.^28^ Primary antibodies were obtained from Cell Signaling Technologies (CST; Danvers, MA, USA), Protein Tech (PT; Rosemount, IL, USA), or Abcam (Ab; Cambridge, MA, USA) and used at the following dilutions: CSE (PT #12217-1-AP, 1:1000), acetylated α-tubulin (Ab #ab24610, 1:1000), α-tubulin (CST #3873, 1:4000), mitofusin 1 (MFN1, PT #13798-1-AP, 1:1000), mitofusin 2 (MFN2, PT #12186-1-AP, 1:1000) Parkin (CST #4211, 1:1000), Phosphatase and Tensin Homolog (PTEN)-induced kinase 1 (Pink1, PT #23274-1-AP, 1:1000), optic atrophy-1 (OPA1, CST #80471, 1:1000) superoxide dismutase 2 (SOD2, CST #13141, 1:1000), BCL2 interacting protein 3 (BNIP3, CST #3769, 1:1000), HDAC6 (CST #7612, 1:1000), autophagy-related protein 5 (ATG5, CST #12994, 1:1000), autophagy-related protein (ATG7, CST #8558 1:1000), light chain 3B (LC3B, CST #2775 1:1000), Unc-51-like kinase (ULK1, CST #8054 1:1000), phosphorylated ULK1 (P-ULK1^Ser757^, CST #6888, 1:1000), GAPDH (PT #10494-1-AP, 1:3000). Anti-mouse (CST #7076, 1:4000) and anti-rabbit (Bethyl Laboratories #A120-102, 1:10 000) secondary antibodies were used as appropriate.

### Statistical analysis

Statistical analyses were performed with GraphPad Prism v9 (La Jolla, CA, USA). Statistical differences for the nerve-stimulated erectile response and the ex vivo vascular reactivity assessments were determined using a 2-way repeated-measures analysis of variance (ANOVA) followed by Tukey’s multiple comparisons post hoc analysis if main effects were detected. Group differences in band intensity density for immunoblot images were determined by 1-way ANOVA followed by Tukey’s multiple comparisons post hoc analysis if main effects were detected. An α-level of 0.05 was used to determine statistical significance in all instances.

## Results

### HDAC6 inhibition augments CC CSE protein content

**Figure 1:**
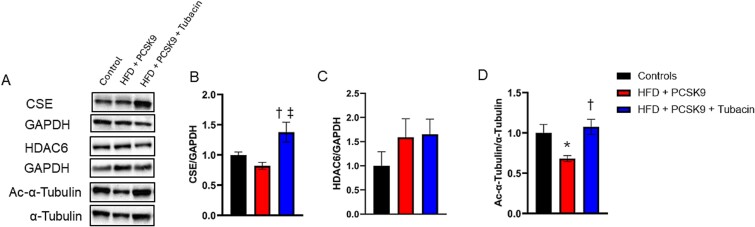
Effect of HDAC6 inhibition on cystathionine γ-lyase (CSE) protein levels in corpus cavernosum tissue. (A) Representative immunoblot images for CSE, acetylated (Ac)-α-tubulin, and their respective loading controls GAPDH and α-tubulin. Quantification of normalized band intensity density of (B) CSE, (C) HDAC6, and (D) Ac-α-tubulin. Data are presented as mean ± SEM for n = 5 mice per group. ^*^P < .05 control vs. HFD + PCSK9 group. ^†^*P* < .05 HFD + PCSK9 group vs. HFD + PCSK9 + tubacin group. ^‡^*P* < .05 HFD + PCSK9 + tubacin group vs. control.

Phenotypic characteristics from these mice have been previously published.^23^ Representative images for immunoblots ran against CSE, HDAC6, and acetylated α-tubulin, as well as their respective loading controls are presented in Figure 1A. Densitometry analysis (Figure 1B) revealed that CSE protein content was elevated in mice treated with tubacin relative to both the control and the HFD + PCSK9 groups. There was a trend for a ~50% increase in HDAC6 protein expression in both HFD + PCSK9 groups relative to the control (Figure 1C), although this trend did not reach statistical significance. As α-tubulin is a well-known HDAC6 substrate, the acetylation status of α-tubulin is a commonly used indirect measure of HDAC6 activity, whereby a decrease in α-tubulin acetylation is indicative of higher HDAC6 activity.^13^^,^^14^ Acetylation of α-tubulin was decreased in the vehicle-treated HFD + PCSK9 mice, which was prevented by tubacin treatment (Figure 1D). Collectively, these results suggest an elevation in HDAC6 activity in the hypercholesterolemic HFD + PCSK9 mouse CC, which was effectively prevented by treatment with the HDAC6 inhibitor tubacin.

### HDAC6 inhibition prevents atherosclerotic erectile dysfunction

**Figure 2:**
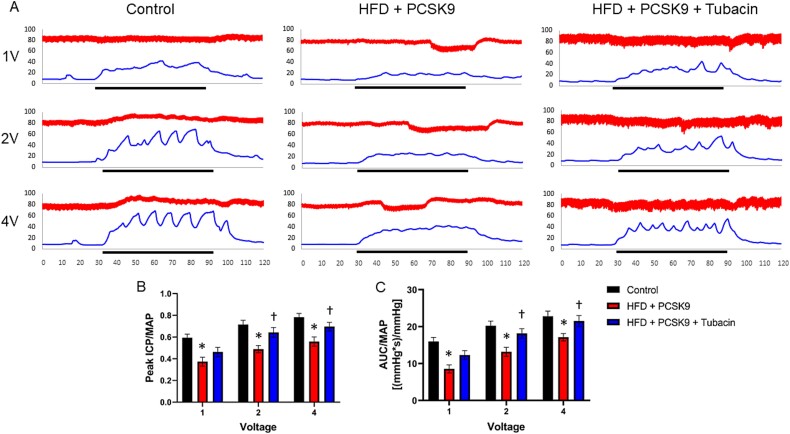
Assessment of cavernous-nerve-stimulated erectile function. (A) Representative tracings of the erectile response at 1, 2, and 4 V of stimulation for 1 mouse in each group. The top tracing represents mean arterial pressure (MAP) obtained from the carotid artery. The bottom tracing represents intracavernous pressure (ICP). The *x*-axis represents time (s). The *y*-axis represents pressure (mmHg). The black line represents the period of electrical stimulation. (B) Erectile function assessed as the peak ICP normalized to MAP. (C) Erectile function assessed as the area under the curve (AUC) for the ICP tracing during electrical stimulation normalized to MAP. Data are presented as mean ± SEM for n = 6 mice per group. ^*^P < .05 control vs. HFD + PCSK9 group. ^†^*P* < .05 HFD + PCSK9 group vs. HFD + PCSK9 + tubacin group.

Representative tracings that include the ICP and MAP readings during cavernous nerve stimulation are presented in Figure 2A. Whether erectile function is assessed as peak ICP/MAP (Figure 2B) or as the AUC/MAP (Figure 2C), erectile function was impaired in the HFD + PCSK9 group throughout the entire voltage range of stimulation. However, the HFD + PCSK9 mice that were treated with tubacin experienced significant restoration of erectile function for both the peak ICP/MAP and AUC/MAP measures in response to 2 and 4 V of electrical stimulation.

### HDAC6 inhibition preserves endothelial function of the CC

**Figure 3:**
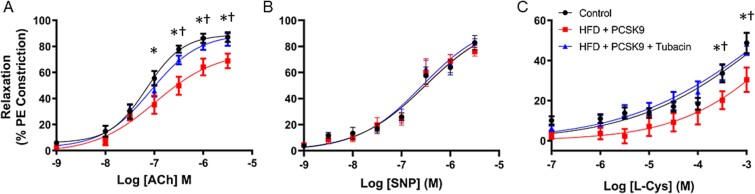
Assessment of agonist-stimulated relaxation of the corpus cavernosum. (A) Endothelium-dependent relaxation was tested with a cumulative dose–response to acetylcholine (ACh). (B) Endothelium-independent relaxation was tested with a cumulative dose–response of sodium nitroprusside (SNP). (C) Relaxation to a cumulative dose–response of the cystathionine γ-lyase substrate L-cysteine (L-Cys). Data are presented as mean ± SEM for n = 7 mice per group. ^*^P < .05 control vs. HFD + PCSK9 group. ^†^*P* < .05 HFD + PCSK9 group vs. HFD + PCSK9 + tubacin group.

Endothelium-dependent relaxation of the CC was significantly impaired in the HFD + PCSK9 mice, as assessed by ACh stimulation (Figure 3A). This effect was prevented in the mice treated with tubacin. Endothelium-independent relaxation, as assessed by relaxation to the nitric oxide donor SNP, was not different among any of the groups (Figure 3B). The relaxation response mediated by the CSE substrate L-cysteine was also impaired in the HFD + PCSK9 mice, an effect that was prevented in the mice treated with tubacin (Figure 3C).

### HDAC6 inhibition effect on markers of mitophagy within the CC

**Figure 4:**
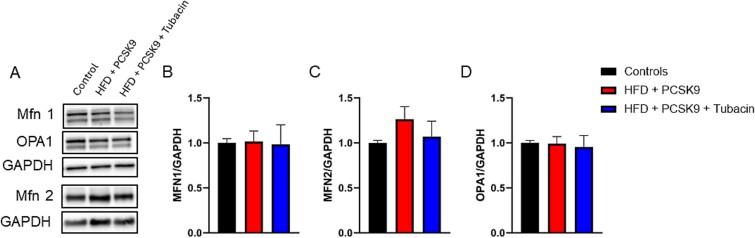
Effect of HDAC6 inhibition on markers of mitofusion protein levels in corpus cavernosum tissue. (A) Representative immunoblot images for MFN1, MFN2, OPA1, and their respective loading controls GAPDH. Quantification of normalized band intensity density of (B) MFN1, (C) MFN2, and (D) OPA1. Data are presented as mean ± SEM for n = 5 mice per group.

**Figure 5:**
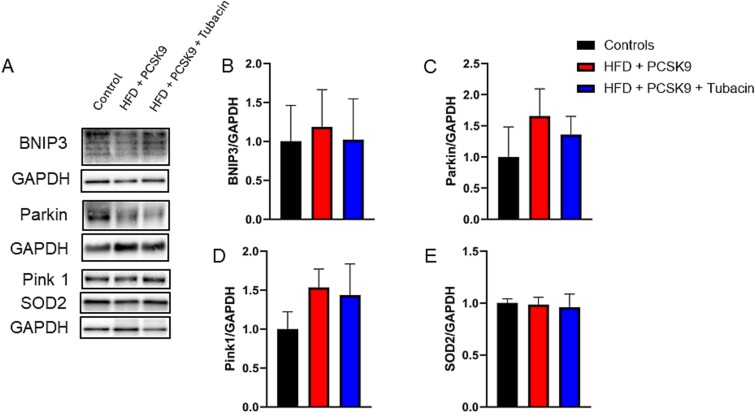
Effect of HDAC6 inhibition on markers of mitophagy protein levels in corpus cavernosum tissue. (A) Representative immunoblot images for BNIP3, Parkin, Pink1, SOD2, and their respective loading controls GAPDH. Quantification of normalized band intensity density of (B) BNIP3, (E) Parkin, (F) Pink1, and (G) SOD2. Data are presented as mean ± SEM for n = 5 mice per group.

Representative immunoblot images for BNIP3, Parkin, MFN2, MFN1, Pink1, OPA1, SOD2, and their respective loading controls GAPDH are presented in Figures 4A and 5A. Densitometry analysis (Figure 4B–D) revealed that no significant difference in MFN1, MFN2, and OPA1 occurred in either the intervention or treatment groups. Similarly, densitometry analysis (Figure 5B–D) revealed that no significant differences in expression of BNIP3, Pink1, Parkin, or SOD2 appeared in either group. Histone deacetylase 6 inhibition did not elicit significant changes in any markers of mitophagy measured compared to control and HFD + PCSK9.

### HDAC6 inhibition effect on markers of autophagy within the CC

**Figure 6:**
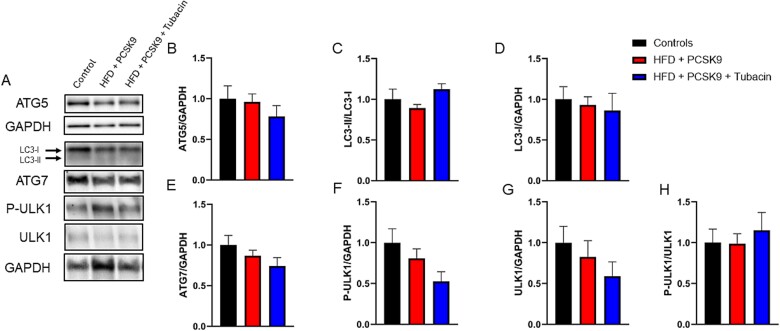
Effect of HDAC6 inhibition on markers of autophagy protein levels in corpus cavernosum tissue. (A) Representative immunoblot images for ATG5, ATG7, LC3B, Ulk1, and their respective loading controls GAPDH and LC3II. Quantification of normalized band intensity density of (B) ATG5, (C) LC3-II/LC3-I ratio, (D) LC3-I, (E) ATG7, (F) P-ULK, (G) ULK1, and (H) P-ULK1 normalized to ULK1. Data are presented as mean ± SEM for n = 5 mice per group.

Representative immunoblot images for ATG7, ATG5, ULK1, LC3B, and their respective loading controls GAPDH are presented in Figure 6A. Densitometry analysis (Figure 6B–H) revealed that no significant difference in the markers of autophagy occurred in the HFD + PCSK9 group. Additionally, no significant difference was found with administration of tubacin compared to both the intervention and control groups.

## Discussion

Erectile dysfunction has become an increasingly prevalent issue across the globe coinciding with the increased rates of obesity, diabetes, and hypercholesterolemia. The current study sought to test the ability of the HDAC6 inhibitor tubacin to ameliorate the decrements in erectile function caused by HFD in mice genetically altered for impaired lipid metabolism. As hypothesized, significant decrements in erectile function as measured by ICP were found in the mice fed an HFD and injected with the PCSK9 AAV. This deterioration of erectile function was restored in the tubacin treatment group. Similarly, in assessment of agonist-stimulated relaxation of the corpus cavernosum both L-cysteine and endothelial-dependent relaxation via acetylcholine were significantly reduced in the HFD + PCSK9 group and significantly restored in the HFD + PCSK9 group treated with tubacin.

The induction of erectile dysfunction in response to a hypercholesterolemic environment has been shown in a multitude of studies most commonly induced via an apolipoprotein E (APO-E) knockout (KO) model as well as with low-density lipoprotein receptor (LDLR)-null mice.^29-32^ Inhibition of HDAC6 via tubacin was able to ameliorate this deterioration at both 2 and 4 V of stimulation. This helps to establish that tubacin via either a protective or restorative pathway can ameliorate the deterioration in erectile function seen with a hypercholesterolemic model. Assessment of agonist-stimulated relaxation of the CC revealed a deterioration in endothelial-dependent relaxation as measured by response to acetylcholine in the HFD + PCSK9 group that was significantly protected or restored in the HFD + PCSK9 group treated with tubacin. This decrease in endothelial-dependent relaxation via acetylcholine in the CC is in agreeance to previous results in hypercholesteremic models performed by Fraga-Silva et al.^33^^,^^34^ This suggests that inhibition of HDAC6 via tubacin administration can provide a protective effect against endothelial dysfunction induced by a hypercholesteremic environment. Endothelial-independent relaxation as measured by SNP showed no significant differences between groups. These results indicate that the endothelium’s ability to produce NO was impaired rather than the underlying smooth muscle responsiveness to NO. It should be noted that treatment of cultured endothelial cells with tubacin has previously been shown to stimulate expression of endothelial NO synthase,^35^ which is another potential explanation for the restorative effects of tubacin that we observed on endothelial function. We also observed a decrement in agonist-stimulated relaxation when the CC was exposed to the CSE substrate L-cysteine. Previous work has found a decrease in aortic relaxation in response to L-cysteine in a db/db diabetic mouse model, which was attributed to an impairment in the CSE/H_2_S pathway.^36^ The protective effect seen with the administration of tubacin to the HFD + PCSK9 group in response to L-cysteine highlights 1 of the potential pathways by which erectile function is preserved. In addition, the significant increase in CSE expression seen with tubacin administration, when compared to both the control and HFD groups, aids to confirm this potential explanation.

This study found no significant changes in expression of protein markers of mitophagy in the CC of the mice in both the intervention and treatment groups. This differs from previous results by Zheng et al., who found that an HFD induced a significant reduction in expression of protein markers of mitophagy in the heart including Pink1, MFN2, and OPA1. Similarly, MFN2 and OPA1 were significantly reduced in the liver and kidneys of the mice fed HFD.^37^ One potential source for this divergence from previous findings is where the samples were taken from; in the current study, CC tissue was used compared to heart, liver, and kidney tissues. Additionally, a recent study investigating HFD and exercise found that both MFN2 and OPA1 expression in skeletal muscle were significantly decreased in mice fed an HFD. Additionally, Pink1 expression was significantly upregulated in mice fed an HFD compared to controls. These markers all showed no significant difference compared to controls when the treatment (exercise intervention) was added.^38^ Conversely, Kang et al. did not find a significant increase in MFN2 with the addition of an HFD alone.^39^ The decline in erectile and endothelial functions suggests a divergence from homeostasis in the CC in this model of hypercholesterolemia. While prior research has suggested that HFD-induced dysregulations in mitophagy and mitochondrial dynamics indicated by changes in these markers contribute to a pathological disruption of cellular homeostasis in these more metabolically active organs, our data fail to suggest that similar dysregulations may account for the functional changes observed in the CC in this model of hypercholesterolemia.

The current study found no significant changes in the measured markers of autophagy in the CC. We hypothesized that the expression of proteins associated with autophagy would be altered due to the oxidative and inflammatory stress associated with the HFD and hypercholesterolemic environment. Markers of autophagy measured in the smooth muscle of the CC in response to metabolic stress has been most extensively done in diabetic animal models.^40-43^ With the diabetic model, LC3-II expression tends to increase in the CC with the exception of Zhu et al. finding no significant difference.^41^ In the current study, no significant differences in LC3-I expression or the LC3-II/LC3-I ratio were found in either the intervention or the treatment group. Zhou et al. found an increase in ATG5 expression using the diabetic model, which was not observed in the current study.^44^ While the increased expression of CC LC3-II suggests an enhanced autophagosome activity in diabetic models, this effect has not previously been investigated with a hypercholesterolemic model. We observed no significant changes in LC3B or ATG5 within the intervention or treatment group.

An et al. observed decreases in expression of total Ulk1 and the LC3-II/LC3-I ratio in the heart tissue of wild-type mice on an HFD and obese Ob/Ob mice on normal chow relative to normal chow-fed wild-type mice. While these changes suggest a decreased autophagic efficiency in the obese heart, no significant changes were observed in the expression of other autophagy markers including ATG3, ATG5, ATG7, ATG12, and P62 in these models.^45^ With Ulk1 shown to play a regulatory role in metabolism of lipids, we hypothesized that corpus cavernosum ULK1 expression would be downregulated in the HFD + PCSK9 groups.^46^ There was a trend toward a decrease in total ULK1 expression in the HFD + PCSK9 group. However, this trend was exacerbated by tubacin treatment, indicating that reversions of possible defects in these autophagic pathways are unlikely to be a contributing factor by which HDAC6 inhibition protects erectile function. There is currently no literature investigating autophagy in the CC in hypercholesterolemic models that highlights the novel aspect of this research. While autophagy does appear to be systemically affected under these circumstances, more work will need to be done to elucidate any potential roles it may play in ED associated with hypercholesterolemia. While we did not observe any significant effects of tubacin treatment on markers of mitochondrial dynamics, mitophagy, or autophagy in the CC, it is important to note that tubacin treatment blunted the increases in aortic stiffness, aortic plaque accumulation, and aortic endothelial dysfunction induced by HFD + PCSK9 in these same mice used in this study.^23^ It is clear that tubacin treatment had some protective effects on endothelial and vascular function in both the central vasculature and erectile tissue. Given the known associations between ED and CVD pathogenesis, further study of systemic HDAC6 inhibition is warranted.

Various HDAC inhibitors have been investigated for their potential to prevent ED in rodent models. The pan-HDAC inhibitor suberoylanilide hydroxamic acid (SAHA) has been found to markedly suppress cavernous TGF-β expression, partially blunt cavernous fibrosis, and partially restore erectile function in a rat model of bilateral cavernous nerve injury (BCNI).^47^ Others have observed similar effects in the BCNI model as well as the partial bladder outlet obstruction model through treatments with valproic acid and sodium butyrate, both of which inhibit class I and IIa HDACs that will not inhibit the class IIb HDAC6.^48^^,^^49^ While it may be tempting to speculate from these results that HDAC6 is minimally involved in the cavernous fibrosis process, HDAC6 has been implicated as an important factor in pulmonary fibrosis.^50^ To date, 4 HDAC inhibitors have undergone clinical trials and subsequent FDA approval for cancer treatment.^51^ The pan-HDAC inhibitor SAHA was the first to receive FDA approval as vorinostat for treatment of T-cell lymphoma.^52^ Currently, there are no FDA-approved HDAC6 inhibitors; however, the selective HDAC6 inhibitor ACY-1215 has shown potential in numerous phase I and II clinical trials with current investigation into use in combinatorial cancer treatment.^53^^,^^54^

A limitation of this study was the single interventional duration investigated. Inclusion of multiple timepoints would provide a stronger picture of the effects of tubacin treatment. Additionally, this study focused strictly on the CC. Investigation of the pre-penile arterial network would provide insight into the vascular function and plaque accumulation in the arteries that may impact erectile function in the hypercholesterolemic state and the potential protective effects of tubacin therein. It is also plausible that protective effects of tubacin on the systemic cardiovascular system, as previously observed in these mice,^23^ positively impacted erectile function. We were also unable to attain sufficient penile tissue to perform histology. As previously discussed, HDAC6 is known to drive fibrosis in other organ systems, and it is possible that tubacin treatment contributed to a preservation of the morphological structure of the erectile tissue in the hypercholesterolemic model. Finally, administration of tubacin to healthy control mice would have provided information on potential adverse impacts on the erectile system, which would impact the potential for clinical translation.

## Conclusion

The potent HDAC6 inhibitor tubacin protects against the reduction in erectile and endothelial functioning induced by a lipid-rich diet in mice that were injected with a gain-of-function adeno-associated virus encoding for PCSK9. Tubacin treatment stimulated CSE transcription in the corpus cavernosum, although the protective effects of HDAC6 inhibition or CSE stimulation do not appear to be mediated by alterations in mitochondrial dynamics, mitophagy, or autophagy. Further investigations are needed to elucidate the method of action by which tubacin can elicit these effects.

## Data Availability

The datasets are available from the corresponding author upon reasonable request.
